# Artificial Intelligence and Integrated Genotype–Phenotype Identification

**DOI:** 10.3390/genes10010018

**Published:** 2018-12-28

**Authors:** Lewis J. Frey

**Affiliations:** 1 Department of Public Health Sciences, Biomedical Informatics Center, Medical University of South Carolina, Charleston, SC 29425, USA; frey@musc.edu or lewis.frey@va.gov; Tel.: +1-843-792-4216; 2 Health Equity and Rural Outreach Innovation Center (HEROIC), Ralph H. Johnson Veteran Affairs Medical Center, Charleston, SC 29401, USA

**Keywords:** artificial intelligence, genotype, phenotype, deep phenotype, data integration, genomics, phenomics, precision medicine informatics

## Abstract

The integration of phenotypes and genotypes is at an unprecedented level and offers new opportunities to establish deep phenotypes. There are a number of challenges to overcome, specifically, accelerated growth of data, data silos, incompleteness, inaccuracies, and heterogeneity within and across data sources. This perspective report discusses artificial intelligence (AI) approaches that hold promise in addressing these challenges by automating computable phenotypes and integrating them with genotypes. Collaborations between biomedical and AI researchers will be highlighted in order to describe initial successes with an eye toward the future.

## 1. Introduction

Genotypes and phenotypes expressed in genomic and phenomic data are related through the processes that converts molecular-scale genotype information into a macroscale manifestation of a particular phenotype of an organism. Integrated multi-omic processes drive this metamorphosis of genomic information stored in the nucleus of the cell. The ability to identify the drivers of this transformation is elusive among the plethora of interacting components that obfuscate our view. Through integrating data into knowledge networks and reasoning over them with artificial intelligence (AI), we can more vividly clarify this transformation.

Alan Turing, in his seminal 1950 paper in the journal *Mind* [1], laid the foundation for the field of AI through framing the task of building and testing machine intelligence using an imitation game, where the machine imitates the interactions of an individual communicating with two players: an adversary and an interrogator. Moreover, he conjectured that discussions about intelligent machines would become commonplace by the end of the millennium through improved computational speed, memory and algorithms.

**Figure 1:**
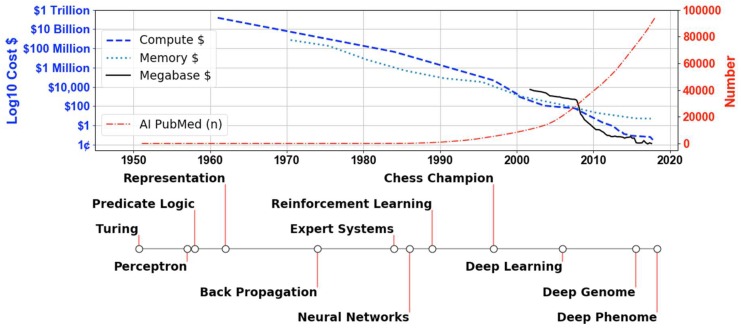
The cost of technology in 2017 US dollars on a log10 scale is plotted in relation to the left axis, and the cumulative number (n) of artificial intelligence (AI) publications in PubMed is plotted in relation to the right axis across time up to and including 2017. The costs of three technologies are compared: Compute, Memory and Megabase. Compute corresponds to the computing costs in gigaflops (one billion floating point operations per second), memory corresponds to the cost of one gigabyte of random access memory, and Megabase corresponds to the cost per megabase sequenced. The cumulative number of PubMed AI-related publications was calculated from identical scripts run for each year starting in 1950. The bottom timeline represents events in the history of AI beginning with Turing’s 1950 publication of “Computing Machinery and Intelligence” and ending with deep learning applied to biomedical phenomic data in 2018 (Supplementary File).

Viewing Figure 1, the cost of computing in gigaflops has gone from tens of billions of dollars in the 1960s to pennies today, with a similar pattern for a gigabyte of random access memory. Combined with such improvements in computing and memory, Turing suggested two avenues of research in algorithms to advance intelligent machines: abstract activity modeling, such as the game of chess; and sensory perception approaches. The timeline in the lower half of Figure 1 is based on algorithmic advances in AI as described in Buchanan’s brief history of AI and is extended with deep learning [2,3]. The labeled events above the timeline in Figure 1 show milestones in abstract activity modeling using predicate logic and knowledge representation approaches resulting in machines being able to imitate and exceed human performance in the game of chess before the year 2000 [2,4,5]. Medical publications related to AI, shown in Figure 1 (thin red dash-dot line) as cumulative counts of PubMed references, have increased following the development of biomedical expert systems such as Mycin [6]. The labeled events below the timeline are those associated with perception approaches such as the perceptron, back propagation, neural networks and deep learning [3,7,8]. Machines imitating and exceeding human performance in the game of Go occurred in 2016 through a combination of both types of approaches: deep learning and tree-based knowledge representation trained through reinforcement learning [9].

The concept of a learning machine was articulated by Turing in terms of evolution, with hereditary material, mutations and natural selection being analogous to the structure of the machine, changes to the machine and performance evaluation providing feedback to the machine at a faster rate than natural selection [1]. In such a framework, machine learning constitutes performance improvement on an evaluation task through the use of labeled data for supervised learning and unlabeled data in unsupervised learning. The speed of reinforcing feedback using deep learning methods over large memory representations of data has made the difference for improving performance on difficult tasks such as object recognition, speech recognition, and playing the game of Go [3,9]. Learning algorithms can miss the mark of the true target, due to the “curse of dimensionality” when too many features in the data space result in overfitting of random variance in the data [10].

Deep learning approaches have managed to navigate the complex space of overfitting and underfitting the data through the use of large amounts of sample and powerful learning algorithms [3]. Deep learning algorithms achieve this high performance through learning multiple layers of non-linear features in the data. Different features are learned depending on initial conditions, making consistent interpretation difficult [11]. The “black box” nature of deep learning approaches highlights differences between the abstraction and perception approaches proposed by Turing to solve the imitation game [1]. The former has well formulated feature representations that are more easily interpreted, but can underfit the data [10]. The latter is not biased to underfit the data, but lacks interpretability due to highly complex features [3,11]. Biomedical research, with the need for biological interpretation, will likely benefit from a hybrid of the two approaches in much the same way as AlphaGo identifies solutions to the game of Go [9]. As can be seen in Figure 1, the solid black line represents the cost per megabase sequenced of genomic information, which went from thousands of dollars to pennies at a faster rate than a gigabyte of random access memory dropped to a few dollars [12,13,14]. Having inexpensive genomic data for building large repositories, that can be integrated and harnessed by these data-hungry AI methods, will have a far-reaching impact on discovery in biomedical domains [15,16,17]. The expertise to integrate and analyze these data resides in both biomedical and AI researchers who have an opportunity to drive a new wave of discovery through high-dimensional analysis of deep genomes and deep phenomes [18,19,20,21].

An important emerging area is the field of Precision Medicine Informatics which takes on the challenges of big data by integrating, in a knowledge network (i.e., a general high-level conceptualization of knowledge represented as facts connected by relationships between facts), multi-omic data on individuals to increase access and discover new knowledge based on a new taxonomy of disease [22,23,24]. Biomedical layers of data at different scales are positioned to be integrated in knowledge networks that can be computationally reasoned over to accelerate discoveries [22,24]. Ontologies can be used to formalize knowledge networks through the description of facts, concepts and properties over which logical reasoning engines can be run to generate new facts or inconsistencies implicit in the ontology [25]. Reasoning over knowledge networks can include AI approaches (e.g., Never Ending Learning) that go beyond function approximation methods to reason over a network of documents through a set of AI modules and measure the consistency of the knowledge learned [26,27].

The gene ontology community has developed a knowledge network of molecular functions, cellular components and biological processes [28]. Human phenotype ontology represents a knowledge network of human disease phenotypes that provide a mechanism for connecting genomic and phenomic medical data [29]. The community managing these ontologies is challenged by scalability issues related to the manual curation of data given the exponential growth in genomic and medical phenomic data. To mitigate these issues, AI methods are needed to automate deep phenotyping in the electronic health record (EHR) by incorporating longitudinal data to improve predictive modeling and integrate phenomic and genomic data [30]. The following will discuss specific examples of AI applied to genomic and phenomic data and how they are making headway against the challenge of exponentially expanding data sets and the goal of advancing scientific knowledge [20,21,31].

## 2. Genomics

The discovery of new treatments will be advanced through understanding the mechanisms by which genomics drives the expression of disease. As indicated, the gene ontology community is iteratively building a knowledge network that can inform biomedical research on the mechanisms and processes that impact the expression of phenotypes [28,32]. The ontology is evaluated on how it performs over time as the ontology is incrementally improved. This is achieved by adding sequences that are annotated with protein function information. These sequences are being added to the system at an exponential rate. However, the validation through experimental findings that confirm or invalidate the protein function information in gene ontology is being added at a linear rate [32]. Thus, there is a vast gap between the number of experimentally validated protein functions and the number of sequences in gene ontology. To address this, there are community-wide evaluation approaches to assess protein function predictions through competitions using a variety of approaches to predict protein function, and to generate candidate predictions at a rate that matches sequence accumulation [28]. Findings on some of the challenges have shown that the use of AI methods, such as the multiple data source k-nearest neighbor algorithm, combined with biological knowledge can give superior results [32].

AI techniques have been used to extract information down to the level of binding properties of genomic sequences that influence the transcriptional networks of cells. Specifically, deep learning methodologies have been used to predict sequence specificity of DNA and RNA binding proteins [20]. The AI approach scanned for motifs in DNA and RNA and identified binding protein promoter sites that would change their binding properties based on variance in single nucleotide polymorphisms, deletions or insertions. The approach identified gain of function mutations or loss of function mutations based on the changing binding affinity of the DNA sequence that the mutations impacted. The deep learning approach is powerful in its ability to discover new knowledge around regulatory processes and biological systems and identifying causal disease variants (i.e., those disease variants that, when changed, change the binding affinities of key regulatory genes in disease processes). The approach also worked on RNA-binding and integration of transcriptomic and genomic analysis [20]. The scale and complexity of data and the techniques now available position AI to be integral to the process of accelerating scientific discovery. The next step is to integrate genomic and phenomic data at different scales.

## 3. Phenomics

AI combined with phenomics can improve disease state detection when the right criteria are used to recognize the drivers. For example, deep learning has been used to identify histological markers of metastatic breast cancer in lymph nodes that pathologists have difficulty identifying, particularly under standard time constraints [31]. For this study, the deep learning algorithm was assessed via an immunochemistry test that verifies whether metastatic cancer was in the tissue or not. The deep learning algorithm performs at an area under the receiver operating characteristic (ROC) curve of 99% compared with 81% for the pathologists who were given about a minute per slide. A strength of deep learning is the capacity to visually identify histopathology phenomics (i.e., phenotypes in histology images) to improve classification of clinically relevant data. Since knowing that breast cancer has metastasized to the lymph node is critical for treatment decisions, the adoption of such AI technologies for decision support will enhance early detection and improve clinical decision making. It also will improve automated phenotype identification from images that will make new genotype and phenotype identification feasible.

Phenomics of EHR data can be developed from formal ontologies of the phenotype criteria. They can also be developed using deep learning to automate phenotype construction for predictive models that distinguish important categories of individuals [29,30]. Deep phenotyping with AI approaches have demonstrated empirically that incorporating temporal information (e.g., lab values over 24 h after hospital admission) into phenomic models improves accuracy of predicting mortality, length of hospital stay and diagnosis at discharge [21]. In this example, deep learning technology was applied to adult EHR data including both laboratory values and clinical notes on a timeline of events prior to hospitalization and for at least 24 h after admission in order to predict their in hospital mortality. The algorithm incorporated time stamped tokens of events in the EHR and used them to improve predictions of mortality. The algorithms predicted in hospital mortality at 93 to 94% area under the ROC curve compared with 91% for existing clinical predictive models [21]. Notably, the analysis was undertaken without the need to harmonize data across multiple hospital centers. The major strength of the AI deep learning approach is the incorporation of temporal information while eliminating the need to curate the phenotype collection manually and harmonize the data manually [21]. The work demonstrates the benefit of incorporate temporal information in patients’ phenotypes, through automated and efficient strategies that show utility in predicting the outcomes of interest, and moves towards an individual focused knowledge network of precision medicine.

## 4. Conclusions

Over 60 years ago, Turing postulated that we would experience a change in perspective on how learning machines are perceived. Breakthrough AI approaches have brought this to pass and have expanded our ability to recognize drivers of phenotypes resulting from single nucleotide variations, valid protein function mechanisms in biological systems, cancer disease states and deep phenotypes automatically constructed from the EHR. Through combining and expanding on these approaches in a collaborative effort, the biomedical community will accelerate discovery and improve our understanding of mechanisms in the genomic and phenomic expression of disease.

## Supplementary Materials

The following are available online at http://www.mdpi.com/2073-4425/10/1/18/s1.
