# Supplementary material for: Artificial Intelligence and Integrated Genotype–Phenotype Identification
Source: Genes (Basel). 2018 Dec 28;10(1):18. doi: 10.3390/genes10010018 (PMC6356893; doi:10.3390/genes10010018)
Supplement: Supplementary file 1 [file genes-10-00018-s001.pdf]

The below script used for PubMed search in Figure 1 was run for each year from 1950 through 2017:

"2017"[Date - Publication] AND (artificial intelligence[Title/Abstract] OR machine learning[Title/Abstract] OR deep learning[Title/Abstract] OR convolutional neural networks[Title/Abstract] OR ai artificial intelligence[MeSH Terms] OR machine learning[MeSH Terms] OR model, neural network[MeSH Terms])

2018 data were excluded from Figure 1 because data for the full year were not available.
